# Supplementary material for: Group B Streptococcus and the vaginal microbiome among pregnant women: a systematic review
Source: PeerJ. 2021 May 17;9:e11437. doi: 10.7717/peerj.11437 (PMC8136278; doi:10.7717/peerj.11437)
Supplement: Supplemental Information 2 [file peerj-09-11437-s002.doc]

**Table S1.** GBS Reported Studies (n = 13)

| **Study** | **Study purpose** | **DNA extraction kit** | **Sequencing method** | **16S rRNA region** | **Taxonomy assignment/classification database** | **N**  **-vaginal specimen/pregnant women** | **Population**  **-participants’ age**  **-country**  **-multi-ethnicity (Y/N)** | **Trimesters** | **Raw data sharing platform (accession number)** |
| --- | --- | --- | --- | --- | --- | --- | --- | --- | --- |
| Fettweis et al. (2014)^9^ | Vaginal microbiome composition variation (in relation with trichomoniasis) | Powersoil® kit (MO BIO Laboratories Inc, Carlsbad, CA) | 16S rRNA gene sequencing | V1-V3 | STIRRUPS classifier (as described in Fettweis et al 2012) | 73/73 | -Age: >18  -USA  -Multi-ethnicity: Y | Not specified  (during pregnancy) | NCBI SRA (project ID phs000256) |
| Ghartey et al. (2014)^10^ | To study the association between E. Coli inhibitory activity and Lactobacillus crispatus/jensenii dominant vaginal microbiomes (of near-term pregnant women and non-pregnant women) | custom protocol | 16S rRNA gene sequencing | V6 | A vaginal microbiome reference library (http://microbiome.fhcrc.org/apps/refpkg/, containing 633 sequences, representing 138 bacterial taxa, using pyNAST) | 8/10 | -Age: 22-41  -USA  -Multi-ethnicity: Y | **○○●**  (35 and 37 wks) | NCBI SRA (Submission ID: SRP034665) |
| Romero et al. (2014)^11^ | Vaginal microbiome composition variation (compare preterm cf. term birth) | custom in house method | 16S rRNA gene sequencing | V1-V3 (27F, 534R) with additional primers for specific taxa and degenerate primer | UCLUST with de novo algorithm and cutoms speciateIT database | 349/90 | -Age: 20-28  -USA  -Multi-ethnicity: Y | **●●●**  (6-40 wks) | NCBI SRA (BioProject PRJNA242473, SRA accession SRA150182, SRP040750) |
| Romero et al. (2014)^12^ | Vaginal microbiome composition variation (compare preg cf. non-preg women) | custom in house method | 16S rRNA gene sequencing | V1-V2 (27F, 338R) | UCLUST with custom community specific reference | 139/22 | -Age: 19-35  -USA  -Women of AA (19), White (2), Hispanic (1) from Detroit MI, USA | **●●●**  (6-40 wks) | NCBI SRA (accession no. SRA026073), metadata in dbGap (dbGap study no. phs000261) |
| Bisanz et al. (2015)^13^ | Oral, gut, vaginal, and milk microbiome composition variation (association with probiotic yogurt and Moringa plant supplement) | PowerSoil-htp 96 Well Soil DNA isolation kit (MoBio) with modifications | 16S rRNA gene sequencing | V4 primers 515F and 806R | Greengenes reference database (May 2013 build) | 42/44 | -Age: 18-40  -Tanzania  -Multi-ethnicity: Y | Not specified  (Mean 32 wks) | N/A |
| MacIntyre et al. (2015)^14^ | Vaginal microbiome composition variation (across pregnancy and postpartum, among British women) | QIAamp DNA Mini kit | 16S rRNA gene sequencing | V1-V2 | RDP MultiClassifier script | 142/42 | -Age: >18  -Multi-ethnicity: Y  -British cohort | **●●●**  (8–12, 20–22, 28–30 and 34–36 wks) | ENA SRA (accession no. PRJEB7703) |
| Kindinger et al. (2017)^15^ | Vaginal microbiome composition variation (in relation with progesterone supplementation, 2^nd^ trimester cervical length, and preterm birth) | QIAamp DNA Mini kit | 16S rRNA gene sequencing | V1-V3 | Silva database (alignment), RDP database (using Wang method), RDP MultiClassifier script and USEARCH (determination of taxonomy assignments) | 161/161 (cross-sectional group) + 234/67 (longitudinal group) = 395/228 | -Age: 18-48 (cross-sectional group)/21-40 (longitudinal group)  -England  -Multi-ethnicity: Y | **○●○**  (cross-sectional: 16 wks) +  **○●●**  (longitudinal: 22, 28, 34 wks) | ENA SRA (accession no. PRJEB11895 and PRJEB12577) |
| Brown et al. (2018)^16^ | Vaginal microbiome composition variation (associate with PPROM, and antibiotics) | QIAamp DNA Mini kit | 16S rRNA gene sequencing | V1-V2 | RDP database (using Wang method), RDP MultiClassifier script and USEARCH (determination of taxonomy assignments) | 165/122 | -Age: not specified  -England  -Multi-ethnicity: Y | **○○●**  (30-37 wks) | ENA SRA (accession no. PRJEB21325) |
| Hočevar et al. (2019)^17^ | Vaginal microbiota and preterm birth (among Slovenian women) | QIAamp DNA mini kit | 16S rRNA gene sequencing | V3-V4 | DADA with RDP classifier to SILVA v128 database and BLAST | 155/155 | -Age: preterm group 31.4 (4.7), term group 30.9 (4.1)  -Slovenian  -White | **○○●**  (after start of labor) | NCBI SRA BioProject (PRJNA544732) |
| Purkayastha et al. (2019)^18^ | Vaginal microbiome composition variation (among women from Northeast India) | custom in house method | 16S rRNA gene sequencing | 27F, 1492R | BLASTn and EZ-taxon | 40/40 | -Age: 18-35  -India | **●●●**  1^st^ (n=12)  2^nd^ (n=16)  3^rd^ (n=12) | NCBI SRA (accession no. in the article Table S2) |
| Romero et al. (2019)^19^ | Vaginal microbiome composition variation (in relation with intra-amniotic infection) | DNeasy PowerLyzer PowerSoil Kit (Qiagen) | 16S rRNA gene sequencing | V4  (515F/806R primers) | Mothur software, classified against SILVA, RDP and BLAST | 8/8 | -Age: 17-38  -USA | **○●●**  (19-37 wks) | N/A |
| Tabatabaei et al. (2019)^20^ | Vaginal microbiota and preterm birth (due to BV) | QIAamp DNA Mini Kit (Qiagen) | 16S rRNA gene sequencing; Primers: 515F and 806R | V4 | GAST (Global Alignment for Sequence Taxonomy) | 450/450 | -Age: <35 (134), >=35 (221)  -Canada  -Multi-ethnicity: Y | **●○○**  (sampled at 1st trimester) | N/A |
| Al-Memar et al. (2020)^21^ | Vaginal microbiota and miscarriages (in 1^st^ and 2^nd^ trimester) | QIAamp DNA Mini kit (Qiagen, Manchester, UK) | 16S rRNA gene sequencing | V1-V2 | RDP database (using Wang method), RDP MultiClassifier script and USEARCH (determination of taxonomy assignments) | 237/161 | -Age: median, (IQR):  1st trimester miscarriage= 34.5 (28.0–38.8)  2nd trimester miscarriage= 31.5 (24.0–35.0)  controls= 34.0 (29.0–38.0)  -England  -Multi-ethnicity: Y | **●●●**  (5–8, 8–10, 10–14, and >14 wks) | ENA SRA (no. PRJEB32479) |

**Note.** The symbol **●○○** indicates the first, **○●○** the second, and **○○●** the third trimester respectively; The symbol ***** indicates GBS was detected and reported from clinical screening test (but not from DNA sequencing); preg = pregnant; AA=African American; RDP=Ribosomal Database Project; ROM=rupture of membranes; PPROM=preterm premature rupture of membranes; SPTB=spontaneous preterm birth; sd=standard deviation; IVF-ET therapy=In Vitro Fertilization & Embryo Transfer therapy; MTCT=mother-to-child transmission; BV=bacterial vaginosis; NCBI = National Center for Biotechnology Information; ENA = European Nucleotide Archive; SRA: Sequence Read Archive/Short Read Archive; no. = number. NCBI BioProject database (<https://www.ncbi.nlm.nih.gov/bioproject>), European Nucleotide Archive (<https://www.ebi.ac.uk/ena/browser>), European Bioinformatics Institute (<http://www.ebi.ac.uk/>), MG-RAST ([http://metagenomics.anl.gov](http://metagenomics.anl.gov/)).

**References**

9. Fettweis JM, Serrano MG, Huang B, Brooks JP, Glascock AL, Sheth NU, et al. An emerging mycoplasma associated with trichomoniasis, vaginal infection and disease. PLoS One. 2014;9(10).

10. Ghartey JP, Smith BC, Chen Z, Buckley N, Lo Y, Ratner AJ, et al. Lactobacillus crispatus dominant vaginal microbiome is associated with inhibitory activity of female genital tract secretions against Escherichia coli. PLoS One. 2014;9(5):1–8.

11. Romero R, Hassan SS, Gajer P, Tarca AL, Fadrosh DW, Bieda J, et al. The Vaginal microbiota of pregnant women who subsequently have spontaneuos preterm labor and delivery and those with a normal delivery at term. Microbiome. 2014;1–15.

12. Romero R, Hassan SS, Gajer P, Tarca AL, Fadrosh DW, Nikita L, et al. The composition and stability of the vaginal microbiota of normal pregnant women is different from that of non-pregnant women. Microbiome. 2014;2(1):1–19.

13. Bisanz JE, Enos MK, PrayGod G, Seney S, Macklaim JM, Chilton S, et al. Microbiota at multiple body sites during pregnancy in a rural tanzanian population and effects of Moringa-supplemented probiotic yogurt. Appl Environ Microbiol. 2015;81(15):4965–75.

14. MacIntyre DA, Chandiramani M, Lee YS, Kindinger L, Smith A, Angelopoulos N, et al. The vaginal microbiome during pregnancy and the postpartum period in a European population. Sci Rep [Internet]. 2015 Aug 11;5(1):8988. Available from: http://www.nature.com/articles/srep08988

15. Kindinger LM, Bennett PR, Lee YS, Marchesi JR, Smith A, Cacciatore S, et al. The interaction between vaginal microbiota, cervical length, and vaginal progesterone treatment for preterm birth risk. Microbiome [Internet]. 2017;5(1):1–14. Available from: http://dx.doi.org/10.1186/s40168-016-0223-9

16. Brown RG, Marchesi JR, Lee YS, Smith A, Lehne B, Kindinger LM, et al. Vaginal dysbiosis increases risk of preterm fetal membrane rupture, neonatal sepsis and is exacerbated by erythromycin. BMC Med. 2018;16(1):1–15.

17. Hočevar K, Maver A, Vidmar Šimic M, Hodžić A, Haslberger A, Premru Seršen T, et al. Vaginal Microbiome Signature Is Associated With Spontaneous Preterm Delivery. Front Med. 2019;6(September):1–12.

18. Das Purkayastha S, Bhattacharya MK, Prasad HK, Upadhyaya H, Lala S Das, Pal K, et al. Contrasting diversity of vaginal lactobacilli among the females of Northeast India. BMC Microbiol. 2019;19(1):1–10.

19. Romero R, Gomez-Lopez N, Winters AD, Jung E, Shaman M, Bieda J, et al. Evidence that intra-amniotic infections are often the result of an ascending invasion - A molecular microbiological study. J Perinat Med [Internet]. 2019 Nov 1 [cited 2020 Sep 5];47(9):915–31. Available from: https://doi.org/10.1515/jpm-2019-0297

20. Tabatabaei N, Eren AM, Barreiro LB, Yotova V, Dumaine A, Allard C, et al. Vaginal microbiome in early pregnancy and subsequent risk of spontaneous preterm birth: a case–control study. BJOG An Int J Obstet Gynaecol. 2019;126(3):349–58.

21. Al-Memar M, Bobdiwala S, Fourie H, Mannino R, Lee YS, Smith A, et al. The association between vaginal bacterial composition and miscarriage: a nested case–control study. BJOG An Int J Obstet Gynaecol. 2020;127(2):264–74.
