# Supplementary material for: Group B Streptococcus and the vaginal microbiome among pregnant women: a systematic review
Source: PeerJ. 2021 May 17;9:e11437. doi: 10.7717/peerj.11437 (PMC8136278; doi:10.7717/peerj.11437)
Supplement: Supplemental Information 3 [file peerj-09-11437-s003.docx]

**Table S2.** GBS Not Reported Studies (n=32)

| **Study** | **Study purpose** | **Analysis level**  **-Species level** | **Streptococcus genus identified**  **(Y/N)** | **DNA extraction kit** | **Sequencing method** | **16S rRNA region** | **Taxonomy assignment/classification database** | **N**  **-vaginal specimen/pregnant women** | **Population**  **-participants’ age**  **-country**  **-multi-ethnicity (Y/N)** | **trimesters** | **Raw data sharing platform (accession number)** |
| --- | --- | --- | --- | --- | --- | --- | --- | --- | --- | --- | --- |
| Dominguez-Bello et al. (2010)^22^ | Delivery mode and newborn initial microbiome (=mother-to-newborn bacterial transmission occurs during birth) | N | Y | PowerSoil® DNA isolation kit (MO BIO laboratories, Carlsbad, CA, USA) | 16S rRNA gene sequencing | V2 | RDP classifier (minimum support threshold: 60%), RDP taxonomic nomenclature | 4/9 | -Age: 21-33  -Venezuela | **○○●**  (sampled 1 hr before delivery) | **N/A** |
| Hernández-Rodríguez et al. (2011)^23^ | Vaginal microbiome composition variation (across pregnancy, among Mexican women) | Species | N | DNAzol reagent (Invitrogen, Carlsbad, Calif, USA) | PCR-denaturing gradient  gel electrophoresis (PCR-DGGE) of 16S rRNA | V3 | BLAST version 2.2.3 search | 64/64 | -Age: 13-43  -Mexico | **●●●** | **N/A** |
| Aagaard et al. (2012)^24^ | Vaginal microbiome composition variation (across pregnancy) | Genus, Species level for Lactobacillus | Y (mentioned Streptococcaceae) | PowerSoil DNA Isolation Kit (MoBio) | 16S rRNA pyrosequencing | V1V3,  V3V5 | RDP with Greengenes database and BLAST | 68/24 | -Age: 18-40  -USA  -Multi-ethnicity: Y | **○●●**  (18-40 wks) | **N/A** |
| Frank et al. (2012)^25^ | Vaginal microbiome composition variation (perinatal MTCT, among HIV-infected pre women) | Y | N | Custom method | 16S rRNA pyrosequencing | Broad-range amplification | BLAST database (All-Species Living Tree Project, ver. LTP_S95) + RDP Naïve Bayesian classifier | 64/64 | -Age: 21-27  -West Africa: Bobo-Dioulasso (Burkina Faso) | **○○●**  (36-38 wks) | GenBank (accession no. JF461543-JF487783) |
| Hyman et al. (2012)^26^ | Vaginal microbiome composition variation (during IVF-ET therapy across different hormonal environments) | Genus, Species level for Lactobacillus | Y | DNeasy kit (Qiagen, Valencia, CA) | 16S rRNA | whole 16S region | RDP database | 99/30 | -Age: 28-45  -USA  -Multi-ethnicity: Y | **●○○**  (6-8 wks) | GenBank (accession no. HQ293151-HQ293203) |
| Hyman et al. (2014)^27^ | Vaginal microbiota and preterm birth | Yes (mostly Genus, Species level only for Lactobacillus) | Y | DNeasy Tissue and Blood Kit (Qiagen,Valencia, California) with some modifications | 16S rRNA gene  (Chain-Terminator (Sanger) Sequencing) | whole 16S region | RDP database | 143/88 | -Age: > 18  -USA  -Multi-ethnicity: Y | **●●●** | GenBank (accession no. JX871219 to JX871316) |
| Walther-António et al. (2014)^28^ | Stable Lactobacillus community during pregnancy | Species level | Y (Supplemental OTU table includes Streptococcus genus, further identified as Streptococcus anginosus and Streptococcus oralis) | MoBio Ultraclean Soil Kit (MoBio Laboratories, Inc., Carlsbad, CA) with some modifications | 16S rRNA | V3V5 | Illinois-Mayo Taxon Operations for RNA Dataset Organization (IM-TORNADO) | 95/12 | -Age: 24-36  -USA | **●●●**  (8–12, 17–21, 27–31, and 36–38 wks) | MG-RAST (sequence IDs: 4563804.3–4563899.3) |
| Baldwin et al. (2015)^29^ | Vaginal/amniotic fluid microbiome composition variation (associate with PPROM) | Genus | N | MoBio Ultraclean Soil Kit with MP Fast Prep | 16S rRNA | V3V5 | BLAST and custom IM-TORNADO | 61/15 | -Age: 19-37  -USA  -Multi-ethnicity: Y | **○●○**  (23-24 wks) | NCBI SRA (study accession no. SRP061714) |
| DiGiulio et al. (2015)^30^ | pregnancy-associated microbiome variation (before and after pregnancy) | Yes | N  (not in top 20) | PowerSoil® DNA isolation kit (MO BIO laboratories, Carlsbad, CA, USA) | 16S rRNA gene sequencing | V3-V5 | RDP classifier version 2.2 | 1188/49 | -Age: 19-45  -USA  -Multi-ethnicity: Y | **●●●**  (weekly sampling) | NCBI SRA (SRP no. 288562) |
| Huang et al. (2015)^31^ | Vaginal microbiome composition variation (sampling sites) | Y (Species level only for Lactobacillus) | Y | DNA MAGNETICS and EXTRACT kit (Shenzhen BioEAsy Biotechnologies. Co., Ltd., China) | 16S rRNA | V4V6 | RDP classifier (default database) | 216/24 | -Age: 19.4-39.2  -China | **●●●** | European Bioinformatics Institute (accession no. from ERS371314 to ERS371619) |
| Brumbaugh et al. (2016)^32^ | Delivery mode and newborn initial microbiome (=mother-to-newborn bacterial transmission occurs during birth) | Genus level | N  *GBS clinical test (+) during prenatal screening | Powerfecal DNA Isolation Kit (Mo Bio, Carlsbad, CA) | 16S rRNA | V1V2 | SINA (1.2.11), Used Silva 115NR99 as reference | 23/23 | -Age: 29-33  -Not specified | **○○●**  (sampled immediately before delivery) | NCBI SRA under (project no. PRJNA278085) |
| Jayaprakash et al. (2016)^33^ | Vaginal microbiome composition variation (following PPROM, to predicts latency duration and perinatal outcomes) | Species level | Y - Streptococcus pseudopneumoniae  identified  *GBS clinical test (+) | magnetic bead-based kit (MagMAX, Life Technologies, Burlington, ON) | Pyrosequencing | cpn60 universal target | cpnDB_nr reference database (downloaded from www.cpndb.ca) | 70/36 | -Age: 22-40  -Canada  -Multi-ethnicity: Y | **○●●**  (24-33 wks) | NCBI SRA (Accession SRP077099, BioProject PRJNA326844) |
| Lauder et al. (2016)^34^ | To examine if bacterial DNA in placenta samples are derive from contamination in dust or commercial reagents | Genus level | Y | PSP Stool DNA Plus kit (STRATEC Biomedical, Berlin-Buch, Germany) | 16S rRNA | V1V2 | UCLUST consensus method of QIIME 1.8, using the GreenGenes 16S rRNA gene database v. 13_8 | 6/6 | -Age: 22-40  -USA  -Multi-ethnicity: Y | **○○●**  (during spontaneous Labor/following ROM) | NCBI SRA (bio project PRJNA309332) |
| Nelson et al. (2016)^35^ | Vaginal microbiome composition variation/profile (high risk for SPTB) | Genus level | Y - also included participants’ BV state | MoBio (Carlsbad, CA) PowerSoilhtp  96 well Soil DNA Isolation plates | 16S rRNA | V4 | Greengenes database (v13_8)  + (BLAST only for Lactobacillus at species level) | 40/40 | -Age: mean 18.74 (sd 2.61)  -USA  -AA women | **●●○**  (<16 wks or 20-24 wks) | **N/A** |
| Subramaniam et al. (2016)^36^ | Vaginal microbiome composition variation (by BV, birth timing & race) | Genus | Y | fecal DNA isolation kit from Zymo Research | 16S rRNA | V4 | RDP classifier with Greengenes database (v13_8) | 39/40 | -Age: mean 21.4 (sd 2.3)  -USA  -Multi-ethnicity: Y | **○●○**  (25-26 wks) | **N/A** |
| Callahan et al. (2017)^37^ | Vaginal microbiota and preterm birth (Caucasian cf. AA) | Yes (only for Lactobacillus and Gardnerella genus  ) | Y  (in Fig. S5-S6.) | PowerSoil® DNA isolation kit (MO BIO laboratories, Carlsbad, CA, USA) | 16S rRNA gene sequencing | V4 | Silva v123 database using implementation of the RDP naive Bayesian classifier | 2179/135 | -Age: 18-45  -USA  -Multi-ethnicity: Y | **●●●**  (weekly sampling) | NCBI SRA (accession no. SRP115697) |
| Freitas et al. (2017)^38^ | Vaginal microbiome composition variation (healthy preg women) | Species level | Y - other species of Streptococcus identified | MagMAX™ Total Nucleic Acid Isolation Kit (Life Technologies, Burlington, ON, Canada) | Pyrosequencing | cpn60 universal target | manually curated reference set of 1,561 OTU sequences representing human vaginal microbiota - generated originally by de novo assembly of cpn60 sequence reads from each of 546 vaginal microbiomes | 182/182 | -Age: 18-49  -USA  -Multi-ethnicity: Y | **●●○**  (11-16 wks) | NCBI SRA (Accession SRP073152, BioProject PRJNA317763) |
| Nasioudis et al. (2017)^39^ | Vaginal microbiome composition variation (first cf. subsequent conception) | Yes (only for Lactobacillus) | Y | QIAamp DNA Mini Kit (Qiagen, Hilden, GER) | 16S rRNA | V1V3 | BLAST | 155/155 | -Age:  first conception 32.3 (3.8); prior termination 33.3 (4.7); spont. abortion 36.0 (5.5); prior delivery 34.6 (3.7)  -USA  -Multi-ethnicity: Y | **●○○**  (8-12 wks) | N/A |
| Nasioudis et al. (2017)^40^ | Vaginal microbiome composition variation (of 1^st^ trimester, stress and autophagy in vaginal epithelial cell) | Yes (Species level for Lactobacillus), Genus level for Gardnerella, Bifidobacterium, and Streptococcus | Y | Custom protocol | 16S rRNA | V1V3 | RDP Bayesian classifier (Ver. 2.5) | 154/154 | -Age: mean 33.8  -USA  -Multi-ethnicity: Y | **●○○**  (8-12 wks) | N/A |
| Roesch et al. (2017)^41^ | Vaginal microbiome composition variation (impact of intrapartum Penicillin prophylaxis for GBS infection among pregnant women with PTB) | Genus level | N  *GBS clinical test (+)    Streptococcus detected in 74% of samples during sequencing (in addition to clinical screening) | QIAamp Fast DNA Stool Mini Kit (Qiagen, Valencia, CA, USA) | 16S rRNA | V4 | QIIME(12) based on the UCLUST method against the Greengenes 13.5 database | 27/27 | -Age: Group 1 (26.2 ± 6.6), Group 2 (25.0 ± 7.0), Group 3 (24.5±4.6), Group 4 (25.7±8.7)  -Brazil | **●●●**  (<32 wks) | NCBI SRA (BioProject ID PRJNA354838, study number SRP093885) |
| Stout et al. (2017)^42^ | Vaginal microbiota and preterm birth (among AA) | Yes (only for Lactobacillus) | Y | PowerSoil® DNA isolation kit (MO BIO laboratories, Carlsbad, CA, USA) | 16S rRNA gene sequencing | V1V3, V3V5 | RDP Naive Bayesian Classifier (ver.2.5, training set 9)  +NCBI database only for Lactobacillus species (BLAST) | 149/77 | -Age: not specified  -USA  -Multi-ethnicity: Y | **●●●** | NCBI Bioproject (no. PRJNA294119) |
| Goltsman et al. (2018)^43^ | pregnancy-associated microbiome (Shotgun metagenomics) | Yes  (strain-level) | N  (not in top 15 Fig.1A/S-Fig.2C) | PowerSoil® DNA isolation kit (MO BIO laboratories, Carlsbad, CA, USA) | Shotgun metagenomics | N/A | EMIRGE | 101/10 | -Age: 20-38  -USA  -Multi-ethnicity: Y | **●●●** | NCBI BioProject database (accession no. PRJNA288562, details in Table S5) |
| Leizer et al. (2018)^44^ | Pregnancy vaginal environments when Lactobacillus are dominant | Y (might be only for Lactobacillus) | N  *GBS clinical test (+) | QIAamp DNA Mini Kit (Qiagen, Hilden, Germany) with some modifications | 16S rRNA | V1-V3 | SILVA bacterial sequence database + RDP Bayesian classifier (2.5) | 157/157 | -Age: 26-44  -Japan | **●○○**  (<12 wks) | **N/A** |
| Matsumoto et al. (2018)^45^ | Vaginal microbiome composition variation (preg cf. non-preg cf. sex workers) | Y  (but not reported, except for Lactobacillus genus) | Y | Custom protocol | 16S rRNA gene sequencing | V3-V4 | RDP Classifier | 24/24 | -Age: non-specified  -Japan | ???  Non-specified | N/A |
| Wylie et al. (2018)^46^ | Vaginal microbiome composition variation (patient vs. provider collected specimen) | Y  (but not reported) | N  (not reported within top 21 genus) | PowerSoil® DNA isolation kit (MO BIO laboratories, Carlsbad, CA, USA) | 16S rRNA | V1V3, V3V5 | RDP naïve bayesian classifier (ver. 2.5) | 94/47 | -Age: not specified  -USA  -Multi-ethnicity: Y | **●●●**  (5-33 wks) | NCBI SRA under Bioproject PRJNA294119, details of the SRA accession no. in article) |
| Chen et al. (2019)^47^ | Vaginal microbiome composition variation (HPV infected preg cf. non-preg) | Yes  (but at least not in the presented 30 in figure 6) | Y  (in most top 20 genus) | QIAamp DNA Mini Kit (Qiagen, Hilden, Germany) | 16S rRNA | V3V4 | RDP classifier script (ver. 2.2), Silva database (Release 128) | 135/86 | -Age: 25-40  -China | **○●●**  (16-30 wks) | NCBI SRA (accession no. SRP126438) |
| Dobbler et al. (2019)^48^ | Vaginal microbiome composition variation (3^rd^ trimester) | Species level, including Lactobacillus, Prevotella, and Gardnerella | N | QIAamp Fast DNA Stool Mini Kit | 16S rRNA | V4 | Greengenes database | 27/27 | -Age: Cluster 1: 27.14 ± 7.4; Cluster 2: 24.33 ± 3.6; Cluster 3: 23.71 ± 6.0  -Brazil | **○○●**  (37-40 wks) | NCBI SRA (accession SRP093885, Run no. SRR7657414 to SRR7657440) |
| He et al. (2019)^49^ | Vaginal microbiota and preterm birth/birth outcome (Chinese women) | Species (only for Lactobacillus genus) | Y | Custom method | 16S rRNA | V3V4 | DADA2 with SILVA and BLAST | 113/113 | -Age: 17-34  -China | **●●●**  (12-27 wks) | NCBI SRA (accession no. SRP216638) |
| Jefferson et al. (2019)^50^ | Vaginal microbiome composition variation (association with Vitamin D) | Species | N | PowerSoil kit (MoBio) | 16S rRNA | V1V3 | STIRRUPS | 537/236 | -Age: 18-42  -USA  -Multi-ethnicity: Y | **●●●**  (7-15, 21-28, and 31-40 wks) | N/A |
| Liu et al. (2019)^51^ | Delivery mode and newborn initial microbiome (=mother-to-newborn bacterial transmission occurs during birth) | Genus | Y | Custom method | 16S rRNA gene | V4V5 | SILVA SSU database v132 | 78/78 | -Age: mean 28.9 (sd 4.5)  -China | **○○●**  (sampled one hour from delivery) | NCBI SRA (accession no. PRJNA559967) |
| Price et al. (2019)^52^ | Vaginal microbiome composition variation (HIV infection & Treatment) | Species level | N | Qiagen ATL buffer (Valencia, CA) with some modifications | Whole genome shotgun (WGS) sequencing | N/A | Alignment using Bowtie2, Taxonomic composition estimated using the HUMAnN2 pipeline | 256/461 | -Age: median 27  <20 (n=24), 20-34 (n=194), >=35 (n=29), missing (n=7)  -Zambiam | **○●○**  (16–20 wks) | N/A |
| Witkin et al. (2019)^53^ | Vaginal microbiome composition variation (according to HDAC1 level in vaginal epithelial cell) | Y (might be only for Lactobacillus genus) | Y | Q"IAamp DNA Mini Kit (Qiagen, Hilden, Germany) with some modifications | 16S rRNA | V1V3 | SILVA bacterial sequence database (ver. 1.27) | 300/150 | -Age: Not specified | **●○○**  (8-12 wks) | N/A |

**Note.** The symbol **●○○** indicates the first, **○●○** the second, and **○○●** the third trimester respectively; The symbol ***** indicates GBS was detected and reported from clinical screening test (but not from DNA sequencing); preg = pregnant; AA=African American; RDP=Ribosomal Database Project; ROM=rupture of membranes; PPROM=preterm premature rupture of membranes; SPTB=spontaneous preterm birth; sd=standard deviation; IVF-ET therapy=In Vitro Fertilization & Embryo Transfer therapy; MTCT=mother-to-child transmission; BV=bacterial vaginosis; NCBI = National Center for Biotechnology Information; ENA = European Nucleotide Archive; SRA: Sequence Read Archive/Short Read Archive; no. = number. NCBI BioProject database (<https://www.ncbi.nlm.nih.gov/bioproject>), European Nucleotide Archive (<https://www.ebi.ac.uk/ena/browser>), European Bioinformatics Institute (<http://www.ebi.ac.uk/>), MG-RAST (<http://metagenomics.anl.gov>).

**References**

1. Dominguez-Bello MG, Costello EK, Contreras M, Magris M, Hidalgo G, Fierer N, et al. Delivery mode shapes the acquisition and structure of the initial microbiota across multiple body habitats in newborns. Proc Natl Acad Sci U S A. 2010;107(26):11971–5.
2. Hernández-Rodríguez C, Romero-González R, Albani-Campanario M, Figueroa-Damián R, Meraz-Cruz N, Hernández-Guerrero C. Vaginal microbiota of healthy pregnant mexican women is constituted by four lactobacillus species and several vaginosis-associated bacteria. Infect Dis Obstet Gynecol. 2011;2011.
3. Aagaard K, Riehle K, Ma J, Segata N, Mistretta TA, Coarfa C, et al. A metagenomic approach to characterization of the vaginal microbiome signature in pregnancy. PLoS One. 2012;7(6).
4. Frank DN, Manigart O, Leroy V, Meda N, Valéa D, Zhang W, et al. Altered vaginal microbiota are associated with perinatal mother-to-child transmission of HIV in African women from Burkina Faso. J Acquir Immune Defic Syndr. 2012;60(3):299–306.
5. Hyman RW, Herndon CN, Jiang H, Palm C, Fukushima M, Bernstein D, et al. The dynamics of the vaginal microbiome during infertility therapy with in vitro fertilization-embryo transfer. J Assist Reprod Genet [Internet]. 2012 Feb 6;29(2):105–15. Available from: <http://link.springer.com/10.1007/s10815-011-9694-6>
6. Hyman RW, Fukushima M, Jiang H, Fung E, Rand L, Johnson B, et al. Diversity of the Vaginal Microbiome Correlates With Preterm Birth. Reprod Sci [Internet]. 2014 Jan 28;21(1):32–40. Available from: <http://journals.sagepub.com/doi/10.1177/1933719113488838>
7. Walther-António MRS, Jeraldo P, Berg Miller ME, Yeoman CJ, Nelson KE, Wilson BA, et al. Pregnancy’s stronghold on the vaginal microbiome. PLoS One. 2014;9(6):1–10.
8. Baldwin EA, Walther-Antonio M, MacLean AM, Gohl DM, Beckman KB, Chen J, et al. Persistent microbial dysbiosis in preterm premature rupture of membranes from onset until delivery. PeerJ. 2015;2015(11):1–18.
9. DiGiulio DB, Callahan BJ, McMurdie PJ, Costello EK, Lyell DJ, Robaczewska A, et al. Temporal and spatial variation of the human microbiota during pregnancy. Proc Natl Acad Sci U S A. 2015;112(35):11060–5.
10. Huang Y-E, Wang Y, He Y, Ji Y, Wang L-P, Sheng H-F, et al. Homogeneity of the Vaginal Microbiome at the Cervix, Posterior Fornix, and Vaginal Canal in Pregnant Chinese Women. Microb Ecol [Internet]. 2015 Feb 18;69(2):407–14. Available from: <http://link.springer.com/10.1007/s00248-014-0487-1>
11. Brumbaugh DE, Arruda J, Robbins K, Ir D, Santorico SA, Robertson CE, et al. Mode of delivery determines neonatal pharyngeal bacterial composition and early intestinal colonization. J Pediatr Gastroenterol Nutr. 2016;63(3):320–8.
12. Jayaprakash TP, Wagner EC, Van Schalkwyk J, Albert AYK, Hill JE, Money DM, et al. High diversity and variability in the vaginal microbiome in women following Preterm Premature Rupture of Membranes (PPROM): A prospective cohort study. PLoS One. 2016;11(11):1–19.
13. Lauder AP, Roche AM, Sherrill-Mix S, Bailey A, Laughlin AL, Bittinger K, et al. Comparison of placenta samples with contamination controls does not provide evidence for a distinct placenta microbiota. Microbiome [Internet]. 2016;4:1–11. Available from: <http://dx.doi.org/10.1186/s40168-016-0172-3>
14. Nelson DB, Shin H, Wu J, Dominguez-Bello MG. The Gestational Vaginal Microbiome and Spontaneous Preterm Birth among Nulliparous African American Women. Am J Perinatol. 2016;33(9):887–93.
15. Subramaniam A, Kumar R, Cliver SP, Zhi D, Szychowski JM, Abramovici A, et al. Vaginal Microbiota in Pregnancy: Evaluation Based on Vaginal Flora, Birth Outcome, and Race. Am J Perinatol. 2016;33(4):401–8.
16. Callahan BJ, DiGiulio DB, Aliaga Goltsman DS, Sun CL, Costello EK, Jeganathan P, et al. Replication and refinement of a vaginal microbial signature of preterm birth in two racially distinct cohorts of US women. Proc Natl Acad Sci U S A. 2017;114(37):9966–71.
17. Freitas AC, Chaban B, Bocking A, Rocco M, Yang S, Hill JE, et al. The vaginal microbiome of pregnant women is less rich and diverse, with lower prevalence of Mollicutes, compared to non-pregnant women. Sci Rep. 2017;7(1):1–16.
18. Nasioudis D, Forney LJ, Schneider GM, Gliniewicz K, France M, Boester A, et al. Influence of Pregnancy History on the Vaginal Microbiome of Pregnant Women in their First Trimester. Sci Rep. 2017;7(1):1–6.
19. Nasioudis D, Forney LJ, Schneider GM, Gliniewicz K, France MT, Boester A, et al. The composition of the vaginal microbiome in first trimester pregnant women influences the level of autophagy and stress in vaginal epithelial cells. J Reprod Immunol [Internet]. 2017;123(March):35–9. Available from: <http://dx.doi.org/10.1016/j.jri.2017.08.009>
20. Roesch LFW, Silveira RC, Corso AL, Dobbler PT, Mai V, Rojas BS, et al. Diversity and composition of vaginal microbiota of pregnant women at risk for transmitting Group B Streptococcus treated with intrapartum penicillin. PLoS One. 2017;12(2):1–13.
21. Stout MJ, Zhou Y, Wylie KM, Tarr PI, Macones GA, Tuuli MG. Early pregnancy vaginal microbiome trends and preterm birth. Am J Obstet Gynecol. 2017;217(3):356.e1-356.e18.
22. Goltsman DSA, Sun CL, Proctor DM, DiGiulio DB, Robaczewska A, Thomas BC, et al. Metagenomic analysis with strain-level resolution reveals fine-scale variation in the human pregnancy microbiome. Genome Res. 2018;28(10):1467–80.
23. Leizer J, Nasioudis D, Forney LJ, Schneider GM, Gliniewicz K, Boester A, et al. Properties of Epithelial Cells and Vaginal Secretions in Pregnant Women When Lactobacillus crispatus or Lactobacillus iners Dominate the Vaginal Microbiome. Reprod Sci. 2018;25(6):854–60.
24. Matsumoto A, Yamagishi Y, Miyamoto K, Oka K, Takahashi M, Mikamo H. Characterization of the vaginal microbiota of Japanese women. Anaerobe [Internet]. 2018;54:172–7. Available from: <https://doi.org/10.1016/j.anaerobe.2018.10.001>
25. Wylie KM, Blankenship SA, Tuuli MG, Macones GA, Stout MJ. Evaluation of patient- versus provider-collected vaginal swabs for microbiome analysis during pregnancy. BMC Res Notes. 2018;11(1):5–11.
26. Chen Y, Hong Z, Wang W, Gu L, Gao H, Qiu L, et al. Association between the vaginal microbiome and high-risk human papillomavirus infection in pregnant Chinese women. BMC Infect Dis. 2019;19(1):1–11.
27. Dobbler P, Mai V, Procianoy RS, Silveira RC, Corso AL, Roesch LFW. The vaginal microbial communities of healthy expectant Brazilian mothers and its correlation with the newborn’s gut colonization. World J Microbiol Biotechnol [Internet]. 2019;35(10):1–14. Available from: <https://doi.org/10.1007/s11274-019-2737-3>
28. He Y, Huang Y, Zhang Z, Yu F, Zheng Y. Exploring profile and potential influencers of vaginal microbiome among asymptomatic pregnant Chinese women. PeerJ. 2019;2019(12):1–17.
29. Jefferson KK, Parikh HI, Garcia EM, Edwards DJ, Serrano MG, Hewison M, et al. Relationship between vitamin D status and the vaginal microbiome during pregnancy. J Perinatol. 2019;39(6):824–36.
30. Liu CJ, Liang X, Niu ZY, Jin Q, Zeng XQ, Wang WX, et al. Is the delivery mode a critical factor for the microbial communities in the meconium? EBioMedicine [Internet]. 2019;49:354–63. Available from: <https://doi.org/10.1016/j.ebiom.2019.10.045>
31. Price JT, Vwalika B, Hobbs M, Nelson JAE, Stringer EM, Zou F, et al. Highly diverse anaerobe-predominant vaginal microbiota among HIV-infected pregnant women in Zambia. PLoS One. 2019;14(10):1–17.
32. Witkin SS, Nasioudis D, Leizer J, Minis E, Boester A, Forney LJ. Epigenetics and the vaginal microbiome: Influence of the microbiota on the histone deacetylase level in vaginal epithelial cells from pregnant women. Minerva Ginecol. 2019;71(2):171–5.
