# Supplementary material for: Group B Streptococcus and the vaginal microbiome among pregnant women: a systematic review
Source: PeerJ. 2021 May 17;9:e11437. doi: 10.7717/peerj.11437 (PMC8136278; doi:10.7717/peerj.11437)
Supplement: Supplemental Information 4 [file peerj-09-11437-s004.docx]

**SEARCH STRATEGY**

We searched three databases (PubMED/MEDLINE, CINAHL, and Web of Science) using two groups of search terms: (“vaginal microbiome” or “vaginal microbiota” or “pregnancy”) and (“16S ribosomal RNA” or “16S rRNA” or “whole genome sequencing”).

***PubMed/MEDLINE Search Strategy***

In PubMed, ((((vaginal microbiome) OR (vaginal microbiota))) AND pregnancy) AND ((16s ribosomal rRNA) OR (16s rRNA) OR (whole genome sequencing) OR (metagenomic)) were entered in the search window with the display option ‘best match’. Limitations of publication date as ‘10 years’, species as ‘humans’, and language as ‘English’ were used.
