# Supplementary material for: Group B Streptococcus and the vaginal microbiome among pregnant women: a systematic review
Source: PeerJ. 2021 May 17;9:e11437. doi: 10.7717/peerj.11437 (PMC8136278; doi:10.7717/peerj.11437)
Supplement: Supplemental Information 5 [file peerj-09-11437-s005.docx]

**Systematic Review and/or Meta-Analysis Rationale**

1. **The rationale for conducting the systematic review / meta-analysis;**

Vaginal microbiome studies frequently report diversity metrics and communities of microbiomes associated with reproductive health outcomes. Reports of *Streptococcus agalactiae* (also known as Group B Streptococcus or GBS), the leading cause of neonatal infectious morbidity and mortality, are notably lacking from the studies of the vaginal microbiome, despite being a known contributor to preterm birth and other complications. Therefore, the purpose of this systematic review was to explore the frequency of GBS reporting in vaginal microbiome literature pertaining to pregnancy and to examine methodological bias that contributes to differences in species and genus-level microbiome reporting. Lack of identification of GBS via sequencing-based approaches due to methodologic or reporting bias may result incomplete understanding of bacterial composition during pregnancy and subsequent birth outcomes.

1. **The contribution that it makes to knowledge in light of previously published related reports, including other meta-analyses and systematic reviews.**

Considerable differences in study design and data formatting methods may contribute to underrepresentation of GBS, and other known pathogens, in existing vaginal microbiome literature. Previous studies have identified considerable variation in methodology across vaginal microbiome studies. This study adds to this body of work because in addition to laboratory or statistical methods, how results and data are shared (e.g., only analyzing genus level data or 20 most abundant microbes), may hinder reproducibility and limit our understanding of the influence of less abundant microbes. Sharing detailed methods, analysis code, and raw data may improve reproducibility and ability to more accurately compare microbial communities across studies.
